# O-Mannosylation of Proteins Enables *Histoplasma* Yeast Survival at Mammalian Body Temperatures

**DOI:** 10.1128/mBio.02121-17

**Published:** 2018-01-02

**Authors:** Andrew L. Garfoot, Kristie D. Goughenour, Marcel Wüthrich, Murugesan V. S. Rajaram, Larry S. Schlesinger, Bruce S. Klein, Chad A. Rappleye

**Affiliations:** a Department of Microbiology, Ohio State University, Columbus, Ohio, USA; b Department of Microbial Infection and Immunity, Center for Microbial Interface Biology, Ohio State University, Columbus, Ohio, USA; c Department of Pediatrics, University of Wisconsin, Madison, Wisconsin, USA; d Departments of Medicine and Medical Microbiology and Immunology, University of Wisconsin, Madison, Wisconsin, USA; Duke University Medical Center

**Keywords:** *Histoplasma*, glycosylation, mannose, phagocyte, thermotolerance

## Abstract

The ability to grow at mammalian body temperatures is critical for pathogen infection of humans. For the thermally dimorphic fungal pathogen *Histoplasma capsulatum*, elevated temperature is required for differentiation of mycelia or conidia into yeast cells, a step critical for invasion and replication within phagocytic immune cells. Posttranslational glycosylation of extracellular proteins characterizes factors produced by the pathogenic yeast cells but not those of avirulent mycelia, correlating glycosylation with infection. *Histoplasma* yeast cells lacking the Pmt1 and Pmt2 protein mannosyltransferases, which catalyze O-linked mannosylation of proteins, are severely attenuated during infection of mammalian hosts. Cells lacking Pmt2 have altered surface characteristics that increase recognition of yeast cells by the macrophage mannose receptor and reduce recognition by the β-glucan receptor Dectin-1. Despite these changes, yeast cells lacking these factors still associate with and survive within phagocytes. Depletion of macrophages or neutrophils *in vivo* does not recover the virulence of the mutant yeast cells. We show that yeast cells lacking Pmt functions are more sensitive to thermal stress *in vitro* and consequently are unable to productively infect mice, even in the absence of fever. Treatment of mice with cyclophosphamide reduces the normal core body temperature of mice, and this decrease is sufficient to restore the infectivity of O-mannosylation-deficient yeast cells. These findings demonstrate that O-mannosylation of proteins increases the thermotolerance of *Histoplasma* yeast cells, which facilitates infection of mammalian hosts.

## IMPORTANCE

For dimorphic fungal pathogens, mammalian body temperature can have contrasting roles. Mammalian body temperature induces differentiation of the fungal pathogen *Histoplasma capsulatum* into a pathogenic state characterized by infection of host phagocytes. On the other hand, elevated temperatures represent a significant barrier to infection by many microbes. By functionally characterizing cells lacking O-linked mannosylation enzymes, we show that protein mannosylation confers thermotolerance on *H. capsulatum*, enabling infection of mammalian hosts.

## INTRODUCTION

For dimorphic fungal pathogens, temperature provides an important cue for both fungal morphology and lifestyle. Elevated temperatures present a restrictive barrier to microbial growth, and mammalian body temperatures have been postulated as one reason for the rarity of fungal pathogens able to cause disease in humans (1, 2). However, for thermally dimorphic fungal pathogens, mammalian body temperature also serves as a cue triggering differentiation into a state better adapted for infection. At lower temperatures (e.g., <30°C), *Histoplasma* grows as saprobic hyphae. At elevated temperatures (e.g., 37°C), *Histoplasma* differentiates into a pathogenic yeast. The ability to transition to the yeast phase and the expression of yeast-phase genes are critical for *Histoplasma* virulence (3, 4). Consequently, a large focus has been on the identification and characterization of yeast phase-specific factors that might facilitate *Histoplasma* pathogenesis (5–12). Many of the extracellular proteins produced by *Histoplasma* yeast cells, but not mycelia, are heavily glycosylated (8), suggesting that glycosylation is important for virulence.

In fungi, mannosylation of proteins is the predominant form of both N-linked and O-linked glycosylation (13). N-linked glycosylation is characterized by the attachment of a large branched glycan structure to the asparagine residue of an N-X-S/T motif of substrate proteins. O-linked glycosylation in fungi is characterized by the attachment of mannose to a serine or threonine residue on substrate proteins, with additional saccharide extension into a linear mannan chain. For N-linked glycans, the extensive branching requires numerous enzymes to build the full glycan (13), while the linear O-linked mannan requires only two or three enzyme families, one for initial mannose attachment and, depending on the organism, one or two for mannan extension (13). For O-linked mannosylation, the initial mannose is attached to the protein by the protein mannosyltransferase (Pmt) family of proteins, with subsequent extension carried out by Ktr and Mnn1 family proteins (*Saccharomyces cerevisiae*) (14) or the Mnt family (*Candida albicans*) (15, 16).

Given the correlation of protein glycosylation with the pathogenic phase of *Histoplasma*, we investigated the functional role of O-linked mannosylation in *Histoplasma* virulence. The *Histoplasma* genome contains genes encoding three Pmt family proteins (Pmt1, Pmt2, and Pmt4), as well as a single Mnt1 homologue. In this study, we characterize *Histoplasma* yeast cells deficient in these glycosylation enzymes. Yeast cells depleted of O-linked mannosylation are viable but are less tolerant of elevated temperatures. We show that these yeast cells have altered cell walls, which changes recognition by immune receptors. *In vivo*, glycosylation-deficient yeast cells are rapidly cleared from the lungs after infection because of loss of the ability to survive at mammalian body temperatures. These findings indicate that O-mannosylation of proteins contributes to *Histoplasma*’s ability to thrive in a mammalian host by increasing the thermotolerance of pathogenic yeast cells.

## RESULTS

### Identification of *Histoplasma* protein mannosyltransferases.

The correlation of extracellular protein glycosylation and *Histoplasma* growth at mammalian body temperature suggests a link between virulence and glycoprotein production (8). To begin investigations into the functional role of the O-glycosylation pathway in *Histoplasma*, we identified three protein-mannosyltransferase homologues (Pmt1, Pmt2, and Pmt4; see Fig. S1A in the supplemental material) and an Mnt1 homologue in the *Histoplasma* transcriptome. Despite the enriched protein glycosylation that characterizes yeast cells, *PMT* genes are transcribed at similar levels in yeast cells and mycelia (17). To probe the function of these enzymes, we isolated a *PMT2* insertional mutant (T-DNA insertion between the third and fourth exons of the *PMT2* gene) and also depleted functions by RNA interference (RNAi). RNAi-based knockdown of each identified gene (via sentinel *gfp* RNAi reduction [18, 19]) indicates at least 90% knockdown of each *PMT* target (Fig. S1B). No major compensatory changes in transcript expression of *PMT1* or *PMT4* was found in the absence of *PMT2* (Fig. S1C), suggesting that the lack of one *PMT* is not compensated for by another, consistent with studies of other fungi (20). Complementation of the *pmt2* mutant with a DNA construct consisting of the native promoter and the *PMT2* gene restored *PMT2* expression (Fig. S1C).

10.1128/mBio.02121-17.1 FIG S1  The *Histoplasma* genome encodes three protein mannosyltransferases. (A) Phylogenetic relationships among fungal gene products with homology to protein mannosyltransferase (Pmt) families. Pmt homologues from *S. cerevisiae* (Sce), *C. albicans* (Cal), *A. fumigatus* (Afu), and *H. capsulatum* (Hca; strain G217B) were identified by BLAST search using defined Pmt proteins from *S. cerevisiae* and *C. albicans*). Protein sequences were aligned by using ClustalW with the BLOSUM62 matrix and assembled into a phylogenic tree. Clades corresponding to the Pmt1 (red), Pmt2 (black), and Pmt4 (blue) families were identified, and *Histoplasma* proteins (boxed) were named according to the closest fungal homologues. (B) Depletion of *PMT* expression by RNAi. *PMT*-*gfp* chimeric RNAi plasmids were transformed into GFP-expressing *Histoplasma* yeast cells, and the depletion of PMT genes was estimated on the basis of the degree of codepletion of GFP sentinel fluorescence. The GFP fluorescence of colonies on solid medium was measured, and fluorescence intensity was determined as a percentage of that of the GFP-expressing parent *Histoplasma* strain. Data represent the mean relative fluorescence, and error bars indicate the standard deviations of replicates (*n* = 4). (C) Expression of putative *PMT* genes in *Histoplasma* yeast cells. Gene expression levels were determined by quantitative reverse transcription-PCR of RNAs harvested from wild-type (*PMT2*), Pmt2-deficient (*pmt2*), and *PMT2*-complemented (*pmt2*/*PMT2*) *Histoplasma* yeast cells. Amplification of PCR products was visualized by SYBR green. Gene expression levels were normalized to the constitutively expressed *RPS15* gene, and the expression was compared to that of *GAPDH* to determine the relative expression level. Error bars indicate the standard deviations of biological replicates (*n* = 3). Asterisks indicate statistically significant differences between strains determined by one-tailed Student *t* test (**, *P* < 0.01; n.s., not significant). Download FIG S1, PDF file, 0.3 MB. Copyright © 2018 Garfoot et al. 2018 Garfoot et al. This content is distributed under the terms of the Creative Commons Attribution 4.0 International license.

### *Histoplasma* protein mannosyltransferases glycosylate extracellular proteins.

**FIG 1:**
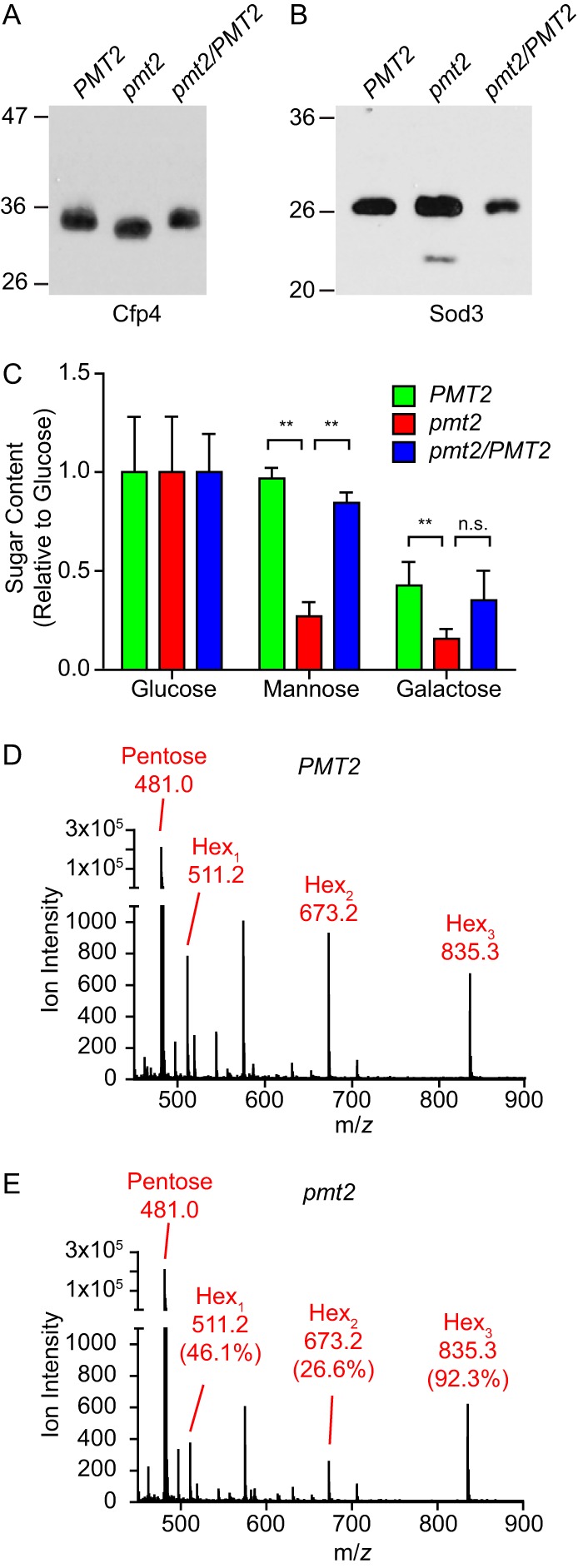
Pmt2 is required for mannosylation of extracellular proteins. Shown are electrophoretic mobility (A and B) and glycosylation (C to E) analyses of proteins secreted by *H. capsulatum* yeast cells. Pmt2-dependent size differences in Cfp4 (A) but not Sod3 (B) proteins were determined by immunoblotting of culture filtrates from wild-type (*PMT2*), Pmt2-deficient (*pmt2*), and complemented (*pmt2*/*PMT2*) *Histoplasma* yeast cells following the removal of *N*-glycans by PNGase F treatment. The molecular masses (in kilodaltons) of protein standards are indicated on the left of the immunoblots. (C) Monosaccharide contents of glycans associated with culture filtrate proteins quantified by GC-MS. The ratio of the abundance of each sugar was calculated relative to the mean value of glucose. Error bars represent the standard deviations, and asterisks indicate statistically significant differences between Pmt-expressing and Pmt2-deficient strains determined by Student *t* test (*n* = 3; **, *P* < 0.01; n.s., not significant). (D and E) Direct measurement of O-linked glycosylation of Cfp4 was determined by quantification of hexose oligomers released from purified Cfp4 by beta elimination and detection of ions corresponding to chains of hexose units by MALDI MS. Shown are the *m/z* values of the major peak and the corresponding hexose chains (Hex_n_). Oligosaccharide ion intensities of Cfp4 from Pmt2-expressing (D) and Pmt2-deficient (E) yeast cells were compared after normalization to an internal ribose standard (pentose). Oligosaccharide abundance is shown as a percentage of that of the wild type.

To confirm the function of *Histoplasma* Pmt proteins in protein glycosylation, secreted proteins were examined for the loss of protein glycosylation by reduction of protein molecular mass. The glycoprotein Cfp4 (8, 21) and β-glucanases Eng1 and Exg8 (8) contain mucin-like domains in the primary amino acid sequence, suggestive of regions potentially modified by O-linked mannosylation (22). Consistent with O-mannosylation of Cfp4, the electrophoretic mobility of the Cfp4 protein is reduced by approximately 2 kDa when produced by Pmt2-deficient yeast cells, and the higher molecular mass is restored by *PMT2* complementation (Fig. 1A). *Histoplasma* Eng1 and Exg8 also have increased electrophoretic mobility in the absence of Pmt2 function (23). We also examined the mobility of two proteins that naturally lack a mucin-like region, secreted proteins Sod3 (12) and Cbp1. Consistent with the lack of O-mannosylation, loss of Pmt2 function did not decrease the molecular mass of either Sod3 (Fig. 1B) or Cpb1 (data not shown). Together, these data show that Pmt2 is necessary for modification of extracellular proteins containing a mucin-like domain.

As further evidence that Pmt2 glycosylates protein, we quantified the saccharide content of extracellular culture filtrate proteins. The protein-associated saccharides from Pmt2-expressing and Pmt2-deficient yeast cells were hydrolyzed into monomeric sugars (e.g., glucose, mannose, and galactose) by acid hydrolysis and quantified by gas chromatography-mass spectrometry (GC-MS). Consistent with Pmt2 catalyzing O-linked mannosylation, there was a significantly smaller amount of mannose (72% reduction) on extracellular proteins from the *pmt2* mutant than on those from the wild type (Fig. 1C). Additionally, loss of Pmt2 caused a 60% reduction in protein-associated galactose. Complementation of the *pmt2* mutant restores both saccharides to wild-type levels.

### Loss of Pmt2 function reduces the glycosylation of Cfp4.

O-linked glycans were removed from purified Cfp4 by beta elimination, and the liberated glycans were characterized by matrix-assisted laser desorption ionization (MALDI) MS (Fig. 1D and E). Spectra for one, two, and three hexose units were identified, and quantitation of the peaks relative to an internal standard showed that glycan levels were 53.9, 73.4, and 7.7% lower, respectively, on Cfp4 protein purified from the *pmt2* mutant than on those from the wild type. As further evidence, electrospray ionization (ESI) MS of the Cfp4 tryptic peptides revealed the mucin-region peptide with variable hexoses (due to glycan fragmentation by ionization) (Fig. S2). The total hexose content on the Cfp4 protein isolated from culture filtrates from Pmt2-deficient yeast cells was lower (23 hexoses; Fig. S2B) than that on Cfp4 from Pmt2-expressing yeast cells (28 hexoses, Fig. S2A). Together, these data confirm that Pmt2 functions in O-glycosylation of extracellular proteins.

10.1128/mBio.02121-17.2 FIG S2  O-linked glycans associated with the mucin-like peptide of Cfp4. Tryptic peptides of Cfp4 produced by *PMT2* wild-type (A) and *pmt2* mutant (B) yeast cells were characterized by capillary-liquid chromatography-nanospray MS on a Thermo Scientific Orbitrap Fusion mass spectrometer to confirm that peptides were glycosylated. Cfp4 was purified from Pmt2-expressing and Pmt2-deficient yeast cells, and peptides were generated by trypsin digestion. Peptides were fractionated by liquid chromatography, and MS_1_ peptides were ionized by electrospray, which variably fragments glycosidic bonds, producing a spectrum of hexose units attached to the peptide. The spectra shown in panels A and B show the mass/charge (*m*/*z*) ratios of the MS_1_ peaks of the Cfp4 mucin-like peptide with variable hexoses. Labels represent the *m/z* ratio of each peak (+4 charge state) and the computed hexose units (Hex_n_). The identity of the peptide (amino acid sequence below) with potential sites of O-linked glycosylation (red) was confirmed by the MS_2_ spectra obtained by electron transfer dissociation fragmentation of the MS_1_ ions. Download FIG S2, PDF file, 0.3 MB. Copyright © 2018 Garfoot et al. 2018 Garfoot et al. This content is distributed under the terms of the Creative Commons Attribution 4.0 International license.

### Loss of O-linked mannosylation alters the yeast surface and yeast recognition by lectin receptors.

**FIG 2:**
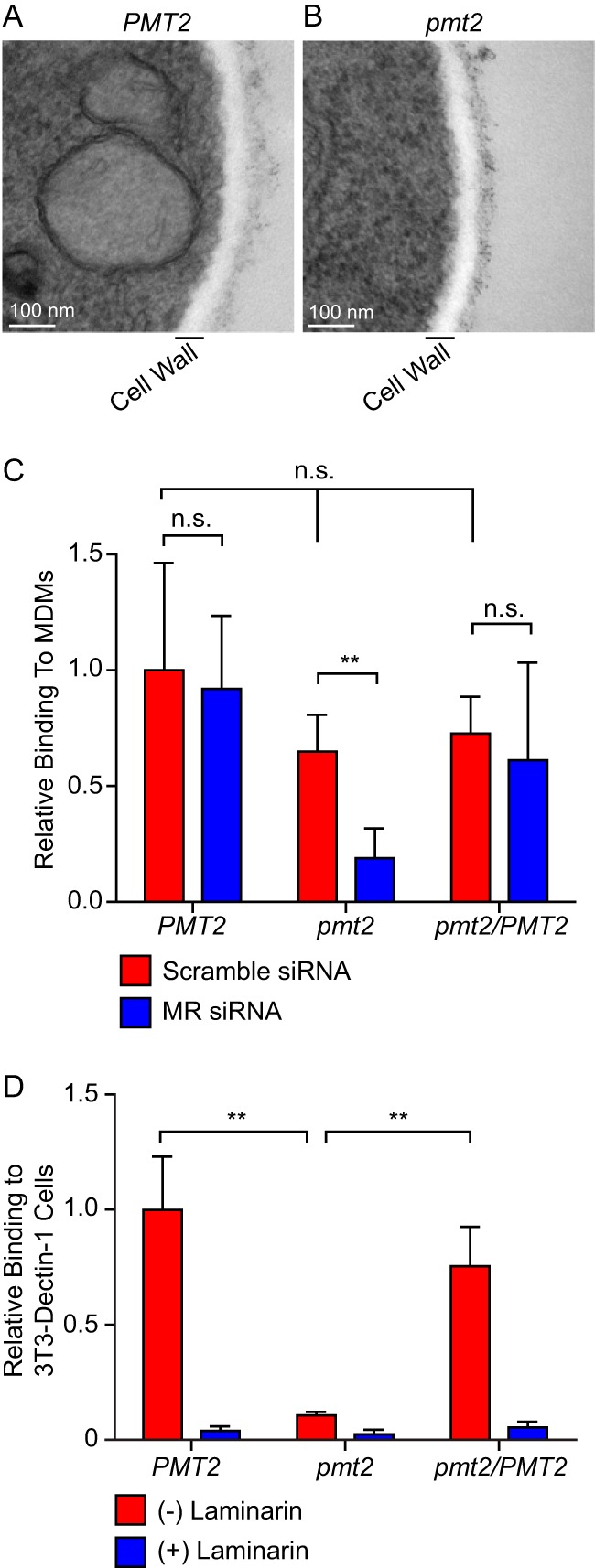
Glycosylation alters the yeast cell surface. (A and B) TEM images of wild-type (*PMT2*; A) and Pmt2-deficient (*pmt2*; B) yeast cells showing the cell wall (electron-translucent region) and surface (electron-dense region surrounding the cell wall). Scale bars represent 100 nm. (C and D) Relative recognition of yeast cells by lectin-type immune receptors. (C) Association of yeast cells with MDMs following siRNA knockdown of MR expression (MR siRNA; blue) or treatment with a control siRNA (scramble siRNA; red). (D) Recognition of *Histoplasma* yeast cells by Dectin-1 determined by quantifying yeast cells bound to Dectin-1-expressing 3T3 fibroblast cells. Association assays were performed in the absence (red) or presence (blue) of 1 mg/ml laminarin to show the specificity of the interaction for Dectin-1 recognition of β-glucans. Associated yeast CFU counts were normalized to the average association of wild-type yeast cells with scramble siRNA-transfected MDMs (C) or binding of wild-type yeast cells to Dectin-1 in the absence of laminarin (D). Error bars represent the standard deviation of replicate assays (*n* = 3), and asterisks indicate statistically significant differences determined by Student *t* test (*n* = 3; **, *P* < 0.01; n.s., not significant).

Since O-linked mannosylation has the potential to alter cell wall proteins, the consequences of the loss of mannosylation for cell wall structure and function were examined. To determine the impact of mannosylation on the *Histoplasma* cell wall, we calculated the 50% inhibitory concentrations (IC_50_s) of the cell wall-binding dyes Uvitex and Congo red (Table S2). Although the *pmt2* mutant, deficient in O-linked mannosylation, was more susceptible to both compounds, the increase in sensitivity was relatively minor (1.5-fold reduction). Furthermore, the IC_50_s of other agents that induce cell wall stress (NaCl and sodium dodecyl sulfate [SDS]) were not affected by the loss of Pmt2 function (Table S2). No major structural defects in the electron-translucent cell wall were detected by electron microscopy of Pmt2-expressing (Fig. 2A) and Pmt2-deficient yeast cells (Fig. 2B) grown in broth culture. Nonetheless, Pmt2-deficient yeast cells consistently showed increased electron density along the periphery of the cell wall, suggesting that Pmt2-dependent mannosylation alters the surface of the cell wall.

Given the important interaction of *Histoplasma* yeast cells with phagocytic cells and the altered cell wall in the absence of O-linked mannosylation, the recognition of yeast cells by immune receptors was tested. Dectin-1 (CLEC7A) and the macrophage mannose receptor (MR/CD206/MRC1/CLEC13D) are two of the major receptors on phagocytes that act as lectins to recognize fungal β-glucans and mannan, respectively (24, 25). To determine if the altered cell surface of Pmt2-deficient yeast cells results in altered recognition by MR, yeast cells were added to human monocyte-derived macrophages (MDMs) with or without small interfering RNA (siRNA)-based depletion of MR (Fig. 2C). While there were no changes in binding to MDMs expressing MR (scramble siRNA), silencing of MR expression decreased the binding of *pmt2* mutant yeast 3.5-fold, demonstrating increased recognition of Pmt2-deficient yeast by MR. To test the recognition of yeast cells by Dectin-1, yeast cells were added to Dectin-1-expressing fibroblasts. In this system, where only a single immune receptor is expressed, recognition of Pmt2-deficient yeast cells is 10-fold lower than that of yeast cells expressing Pmt2 (Fig. 2D). Competitive inhibition with soluble β-glucan eliminates the binding of Pmt2-deficient yeast cells, showing that binding is due to the recognition of cell wall β-glucans. Together, these data indicate that O-linked mannosylation alters yeast cell recognition by individual host lectin-type receptors; however, there is no overall change in the total association of yeast cells with phagocytes (Fig. 2C, scramble siRNA cells; data not shown).

### *Histoplasma* survival *in vivo* requires protein mannosylation.

**FIG 3:**
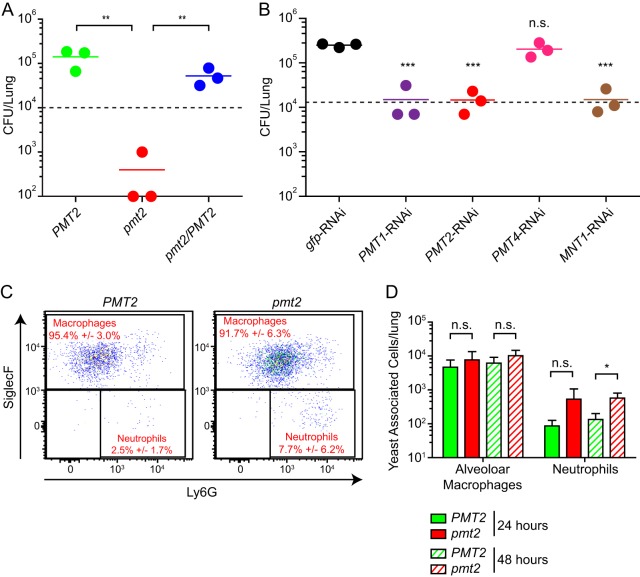
O-linked protein mannosylation is required for *Histoplasma* survival *in vivo*. (A and B) *Histoplasma* burdens in mouse lungs 3 days following respiratory infection. Mice were infected intranasally with wild-type, *pmt2*, and *pmt2*/*PMT2 Histoplasma* yeast cells (A) or wild-type (*gfp* RNAi) or *PMT1*, *PMT2*, *PMT4*, and *MNT1* RNAi *Histoplasma* yeast cells (B). Data points represent the numbers of CFU from individual mouse lungs (*n* = 3), and horizontal bars indicate the mean fungal burdens. Dashed horizontal lines indicate the inoculum doses. (C) *Histoplasma*-associated lung cell populations 24 h after *PMT2* or *pmt2 Histoplasma* infection characterized by flow cytometry. Percentage represents the mean ± the standard deviation of replicates (*n* = 5) of yeast-associated phagocytes, alveolar macrophages (SiglecF^+^), and neutrophils (SiglecF^−^ Ly6G^+^). (D) Numbers of yeast cell-associated alveolar macrophages (SiglecF^+^ CD64^+^ CD11C^+^) and neutrophils (SiglecF^−^ Ly6G^+^) in mouse lungs at 24 h (solid shading) or 48 h (hatched shading) following respiratory infection with *PMT2* (green) or *pmt2* mutant (red) yeast cells. Error bars represent the standard deviations of replicate infections (*n* = 5). Asterisks indicate statistically significant differences between wild-type and mutant strains determined by Student *t* test (n.s., not significant; **, *P* < 0.01; ***, *P* < 0.001).

To determine the functional role of O-linked mannosylation in the pathogenesis of *Histoplasma*, we examined the virulence of *Histoplasma* yeast cells lacking Pmt functions in a sublethal model of respiratory histoplasmosis. In contrast to wild-type yeast cells, whose lung fungal burden increased 20-fold over 3 days of infection, Pmt2-deficient *Histoplasma* yeast cells were strikingly reduced to levels below that of the inoculum (Fig. 3A). RNAi-based depletion of Pmt1 and Pmt2 similarly reduced lung infection 16-fold; however, depletion of Pmt4 had no effect on virulence (Fig. 3B). Depletion of Mnt1, which acts downstream of Pmt functions, caused a nearly identical reduction (16-fold) in lung fungal burdens, providing further evidence that O-mannosylation of proteins is required for *Histoplasma* pathogenesis (Fig. 3B).

To gain insight into the nature of the attenuation of *Histoplasma* Pmt2-deficient yeast cells early in infection, we investigated the phagocytes associated with *Histoplasma* yeast cells in the lungs. Mice were infected with fluorescently labeled *Histoplasma* yeast cells, and the lung phagocyte populations with fluorescent yeast cells were characterized by flow cytometry at 24 and 48 h postinfection. After 24 h of infection, >90% of the yeast cells were associated with alveolar macrophages, with a small amount of yeast cells associated with neutrophils (Fig. 3C and D). Pmt2-expressing and Pmt2-deficient yeast cells showed similar phagocyte association profiles (although there was a 3-fold increase in the number of neutrophils associated with *pmt2* mutant yeast cells, the difference did not reach statistical significance; Fig. 3D). At this early time point, yeast cells were not significantly associated with monocytes, conventional dendritic cells, or monocyte-derived dendritic cells (i.e., Ly6G^−^ SiglecF^−^ cells; Fig. 3C). At 48 h postinfection, similar trends were found. The 3-fold increase in neutrophils associated with *pmt2* mutant yeast cells was statistically significant; however, the total neutrophil population increased 4-fold at 48 h (Fig. S3) with no change in the number of yeast-associated neutrophils. These data indicate that loss of O-linked mannosylation does not substantially alter the association of yeast cells with the major phagocytic cell populations in the lung.

10.1128/mBio.02121-17.3 FIG S3  Yeast-associated phagocyte populations in the lung 48 h after infection. Total numbers of *Histoplasma*-associated phagocytes were determined by flow cytometry with surface markers for alveolar macrophages (SiglecF^+^ CD64^+^ CD11C^+^) and for neutrophils (SiglecF^−^ Ly6G^+^). Lungs were harvested from mice 24 or 48 h after infection with *PMT2* (green) or *pmt2* (red) yeast cells (prestained with Uvitex) and single cell suspensions of lungs. Error bars represent the standard deviations of replicate infections (*n* = 5). Asterisks indicate statistically significant differences between wild-type and mutant strains determined by Student *t* test (n.s., not significant; *, *P* < 0.05). Download FIG S3, PDF file, 0.3 MB. Copyright © 2018 Garfoot et al. 2018 Garfoot et al. This content is distributed under the terms of the Creative Commons Attribution 4.0 International license.

### Control of Pmt2-deficient yeast cells *in vivo* is independent of macrophages and neutrophils.

**FIG 4:**
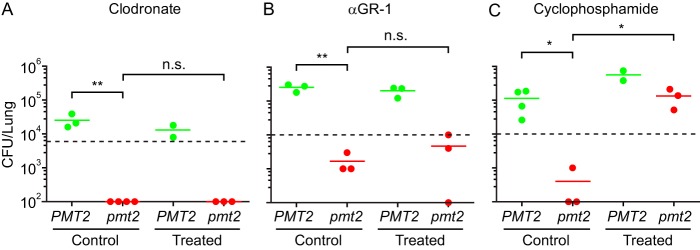
Control of *pmt2* mutant yeast cells is not mediated by phagocytes. (A to C) Control of *pmt2* mutant yeast cells after depletion of phagocytes. Mice were depleted of alveolar macrophages (A; liposomal clodronate treatment) or neutrophils (B; anti-GR-1 antibody treatment) prior to intranasal infection with wild-type (green) or *pmt2* mutant yeast cells (red). (C) Immunosuppression by cyclophosphamide treatment. Control groups were treated with a vehicle control prior to infection. Lungs were harvested 3 days following infection, and fungal burdens were determined by CFU enumeration. Data points represent the CFU counts of individual mouse lungs (*n* = 3), and horizontal bars indicate the mean fungal burdens. Horizontal dashed lines indicate the inoculum doses. Asterisks indicate statistically significant differences between strains determined by Student *t* test (*n* = 3; n.s., not significant; *, *P* < 0.05; **, *P* < 0.01).

Since the host cells associated with *Histoplasma* yeast cells *in vivo* were macrophages and neutrophils and O-linked mannosylation affects immune receptor recognition of yeast cells, we tested whether the lack of Pmt-dependent mannosylation affected the survival of yeast cells in these cell types. Surprisingly, Pmt2-deficient yeast cells are as viable as wild-type yeast cells following infection of either primary macrophages or neutrophils (Fig. S4). To test if macrophages or neutrophils mediated the reduced fungal burdens of Pmt-deficient yeast cells *in vivo*, we depleted mice of phagocytes prior to infection. Administration of liposomal clodronate (intranasal) reduced the number of alveolar macrophages by approximately 70 to 80% (Fig. S5A). This depletion of macrophages did not significantly affect host infection by wild-type yeast cells and importantly did not rescue the lung infectivity of Pmt2-deficient yeast cells, indicating that macrophages are not the primary source of control of the *pmt2* mutant (Fig. 4A). Administration of anti-GR-1 antibody to mice caused a 95% lower level of circulating neutrophils than in mice treated with a control antibody (Fig. S5B). As with macrophages, depletion of neutrophils did not restore the survival of Pmt2-deficient yeast cells in murine lungs (Fig. 4B). In addition to neutrophils, anti-GR-1 antibody also depletes inflammatory monocytes (26), indicating that inflammatory monocytes are also not the source of immune control of yeast cells lacking O-linked mannosylation. In contrast to depletion of individual phagocyte populations, immunosuppression of mice by administration of cyclophosphamide (90% depletion of circulating white blood cells; Fig. S5C) results in full recovery of the *in vivo* fitness of Pmt2-deficient yeast cells (Fig. 4C).

10.1128/mBio.02121-17.4 FIG S4  Survival of Pmt2-deficient yeast cells in primary mammalian phagocytes. Murine peritoneal macrophages or human neutrophils were infected with wild-type (green; *PMT2*), Pmt2-deficient (red; *pmt2*), or complemented (blue; *pmt2*/*PMT2*) *Histoplasma* yeast cells at an MOI 1:10 (yeast-to-phagocyte ratio). Peritoneal macrophages were obtained from C57BL/6 mice by peritoneal lavage and seeded into 96-well tissue culture plates at 2 × 10^4^/well in Dulbecco’s modified Eagle medium (DMEM) supplemented with 10% fetal bovine serum at 37°C. PMNs (neutrophils) were isolated from human peripheral blood by Ficoll density sedimentation, followed by dextran 500 sedimentation of erythrocytes. Neutrophils were seeded into 96-well tissue culture plates at 2 × 10^5^/well in DMEM with 10% autologous human serum. Phagocytic cells were allowed to adhere at 37°C prior to the addition of yeast cells. Viable yeast cells were determined after 8 h by the phagocytes were lysed with water and the lysate was plated on solid medium for CFU enumeration. Survival was determined by comparison of the CFU count to that of yeast cells cultured in DMEM in the absence of phagocytes. Error bars represent the standard deviations of replicate assays (*n* = 3), and asterisks indicate statistically significant differences between strains determined by Student *t* test (*n* = 3; n.s., not significant). Download FIG S4, PDF file, 0.3 MB. Copyright © 2018 Garfoot et al. 2018 Garfoot et al. This content is distributed under the terms of the Creative Commons Attribution 4.0 International license.

10.1128/mBio.02121-17.5 FIG S5  Phagocyte depletion *in vivo*. Depletion of lung macrophages (A) and neutrophils (B and C) at days 0 (D0) and 3 (D3) relative to the time of *Histoplasma* infection (corresponding to 24 and 96 h following initial treatment at day −1). (A) To deplete lung macrophages, mice were administered a single dose of Clodrosome (CLD; 250 µg) or control lipid (250 µg) intranasally on day −1. (B) Neutrophils were depleted by i.p. injection of anti-GR-1 antibody (Ly6C/Ly6G clone RB6-8C5; 500 µg) or control antibody (anti-KLH clone LTF2; 500 µg) every 48 h (i.e., days −1 and 1). (C) Immune cells were depleted by i.p. injection of cyclophosphamide (CTX; 150 mg/kg) or an equal volume of saline (PBS) on day −1. Immune cells were enumerated by collection of alveolar lavage fluid (A) or peripheral blood (B and C), preparation of fluid or blood smears, and staining of slides with Wright-Giemsa for visualization by microscopy. The average number of immune cells per field of view (*n* = >20) was calculated for each mouse. Data represent the average ± the standard deviation of replicate treatments (*n* = 3). Asterisks indicate statistically significant differences between control and treated mice determined by Student *t* test (**, *P* < 0.01; ***, *P* < 0.001). Download FIG S5, PDF file, 0.2 MB. Copyright © 2018 Garfoot et al. 2018 Garfoot et al. This content is distributed under the terms of the Creative Commons Attribution 4.0 International license.

### O-mannosylation enables yeast tolerance of elevated temperatures.

**FIG 5:**
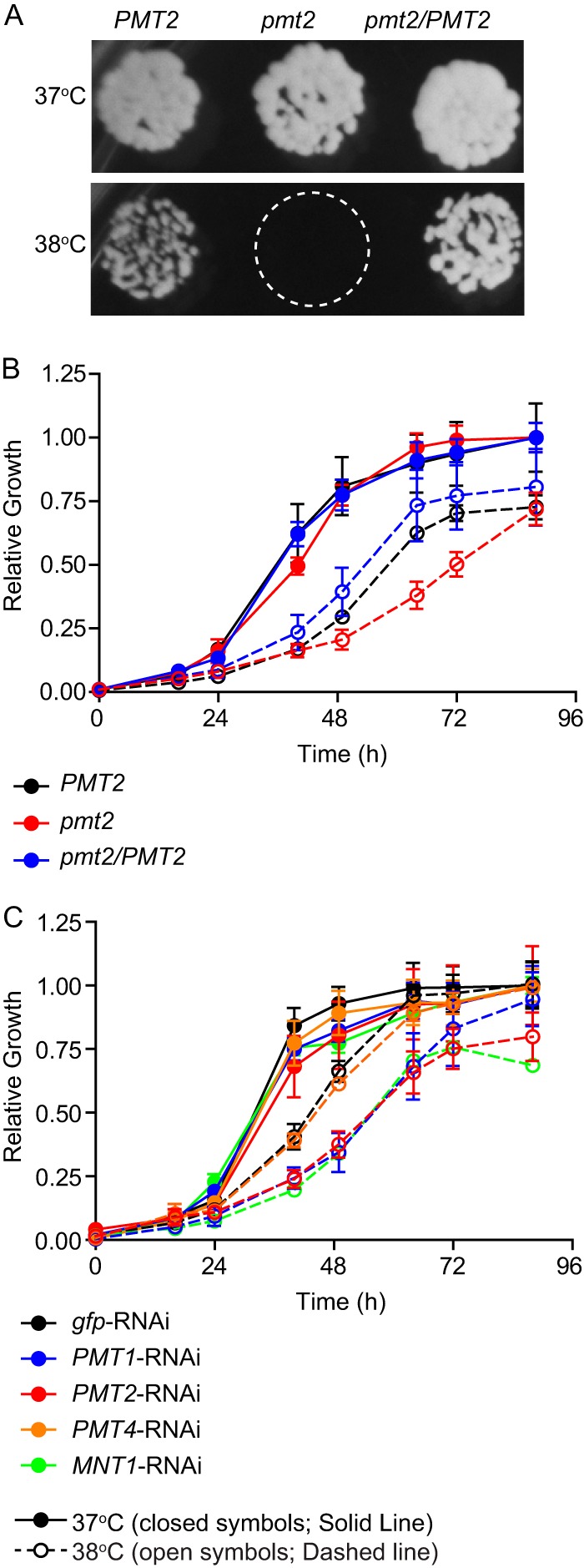
Yeast cells lacking O-linked mannosylation have reduced thermotolerance. (A) Growth of *Histoplasma* yeast cells on solid medium at elevated temperatures. Representative images (*n* = 3 biological replicates) of wild-type, *pmt2*, and *pmt2*/*PMT2 Histoplasma* yeast cells grown at 37 or 38°C are shown. (B and C) Growth rates (monitored by measuring the OD_595_) of O-mannosylation-deficient strains in liquid medium at 37°C (solid lines with filled symbols) and 38°C (dashed lines with open symbols). (B) Growth of wild-type (*PMT2*) (black), *pmt2* (red), and *pmt2*/*PMT2* (blue) yeast cells. (C) Growth of *gfp* (black), *PMT1* (blue), *PMT2* (red), *PMT4* (orange), and *MNT1* (green) RNAi strains. Relative growth was determined by comparison to growth at 37°C. Error bars represent the standard deviations of replicate tests (*n* = 3).

Pmt2-deficient yeast cells are viable at 37°C, but growth at elevated temperatures requires O-linked mannosylation of proteins. On solid medium *in vitro*, the growth of Pmt2-expressing and Pmt2-deficient *Histoplasma* yeast cells is the same at 37°C (Fig. 5A). Increasing the temperature to 38°C slightly impaired the growth of Pmt2-expressing yeast cells; however, the growth of Pmt2-deficient yeast cells was virtually eliminated (Fig. 5A). In broth culture at 37°C, Pmt2-deficient yeast cells grew with the same kinetics as Pmt2-expressing strains (Fig. 5B). At 38°C, the growth rate of all yeast cells decreased; however, the *pmt2* mutant was even further attenuated (Fig. 5B). Strains depleted of Pmt1 and Mnt1 are similarly impaired at 38°C compared to 37°C, but depletion of Pmt4 function did not impair growth (Fig. 5C), paralleling the *in vivo* virulence defects (Fig. 3B). These results indicate that Pmt1- and Pmt2-dependent O-linked mannosylation, but not Pmt4-initiated glycan addition, increased *Histoplasma* survival of heat stress.

**FIG 6:**
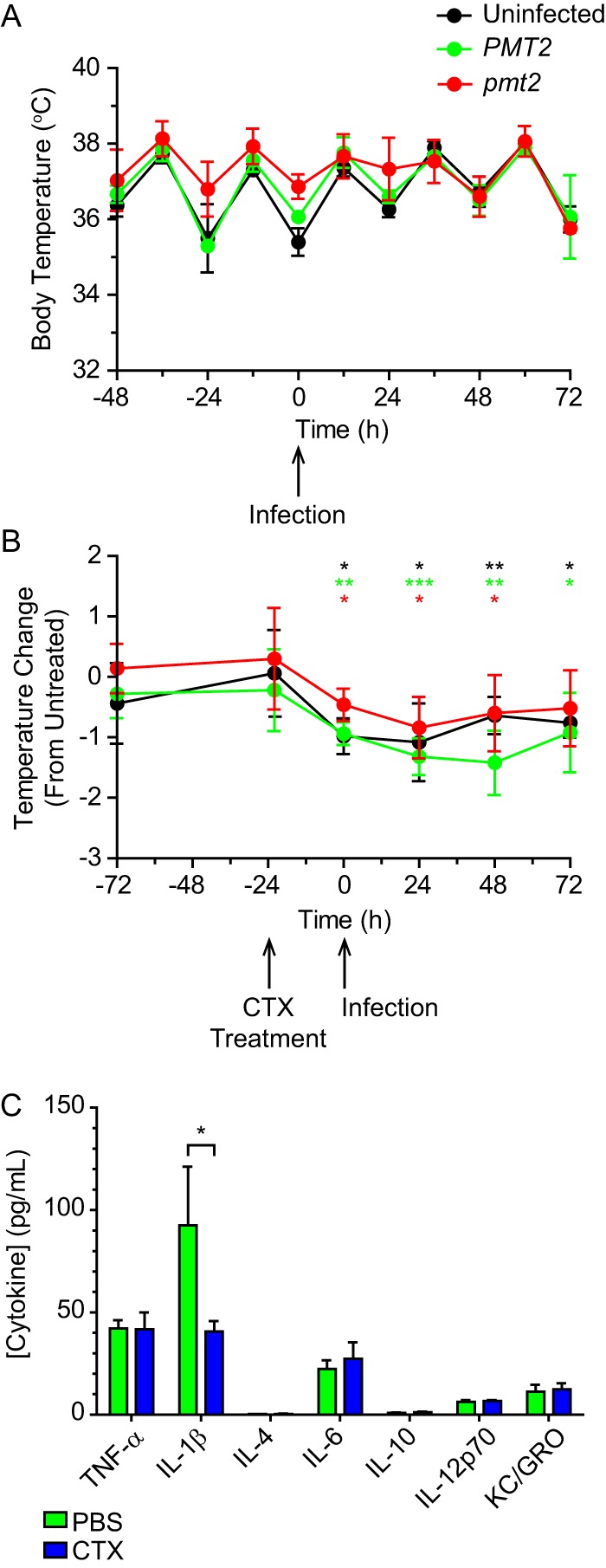
Reduction of core body temperature allows survival of *pmt2* mutant yeast cells. (A) Oscillation of murine core body temperature before and after respiratory infection with *Histoplasma* yeast cells. The rectal temperatures of uninfected mice (black) and mice infected with wild-type (green; *PMT2*) or Pmt2-deficient (red; *pmt2*) yeast cells were measured every 12 h (the −48-h time point was 1100 h). (B) Effect of cyclophosphamide treatment on the core body temperature of mice. Mice were treated with cyclophosphamide (CTX) 24 h prior to infection with wild-type (green; *PMT2*) or Pmt2-deficient (red; *pmt2*) yeast cells, and their rectal temperatures were monitored over time. Data represent the temperatures of uninfected or infected mice relative to those of mice given the control treatment (PBS). Time zero is the time of infection. (C) Lung cytokine levels measured after CTX treatment. Mice were treated with either PBS or CTX 24 h prior to infection with either *PMT2* (green line) or *pmt2 Histoplasma* yeast cells, and lung homogenate was harvested 24 h after infection. Error bars represent the standard deviations of animals (*n* = 3), and asterisks indicate statistically significant differences from untreated control mice (B and C) determined by Student *t* test at each time point (*n* = 5; *, *P* < 0.05; **, *P* < 0.01; ***, *P* < 0.001).

Since yeast cells deficient in O-linked mannosylation are sensitive to elevated temperatures, we tested if temperatures encountered during mammalian infection are the source of the decreased *in vivo* fitness of Pmt2-deficient yeast cells. Like other mammals, the core body temperature of mice varies with activity; the baseline core temperature of mice averaged 36.5°C during daytime (when mice are less active) but increased to 37.8°C during the night (when mice are active) (Fig. 6A). Surprisingly, sublethal infection with either Pmt2-expressing or Pmt2-deficient yeast cells did not change core body temperatures (Fig. 6A), despite the production of fever-inducing cytokines (e.g., tumor necrosis factor alpha [TNF-α], interleukin-1β [IL-1β], and IL-6) after infection (Fig. S6). Although infection did not produce a febrile response in host animals (mice maintained a daily average body temperature of 37°C), the roughly 1°C normal increase during periods of activity is sufficient to cause restrictive conditions for Pmt2-deficient yeast cells (Fig. 5A).

10.1128/mBio.02121-17.6 FIG S6  Loss of O-linked mannosylation does not alter the cytokine response to *Histoplasma* yeast cells. C57BL/6 mice were infected intranasally with 2 × 10^4^ Pmt2-expressing (green; *PMT2*) or Pmt2-deficient (blue; *pmt2*) yeast cells, and their lungs were collected at 24 h postinfection. Lung cytokine levels were determined by analysis of lung homogenates with a proinflammatory multiplex cytokine assay kit (Mouse Proinflammatory Panel 1; Meso Scale Diagnostics). Error bars represent the standard deviations of replicate infections (*n* = 3). Download FIG S6, PDF file, 0.3 MB. Copyright © 2018 Garfoot et al. 2018 Garfoot et al. This content is distributed under the terms of the Creative Commons Attribution 4.0 International license.

As Pmt2-deficient yeast cells have less thermotolerance and cyclophosphamide treatment fully restored the virulence of *pmt2* mutant yeast cells (Fig. 4C), we investigated if the cyclophosphamide-mediated rescue was linked to the host animal body temperature. Independent of infection with *Histoplasma*, cyclophosphamide treatment of mice reduced the relative body temperature by an average of 1°C (during both the day and night oscillations) as early as 24 h after treatment (Fig. 6B) and the relative reduction persisted throughout the infection time period. The reduced body temperature following cyclophosphamide treatment correlated with reduced production of proinflammatory IL-1β (Fig. 6C), which can affect the core body temperature (27). The cyclophosphamide-induced drop in the core body temperature and rescue of the pmt2 mutant yeast cells’ virulence indicate that the attenuation seen stems from reduced thermotolerance in the absence of Pmt-catalyzed O-linked protein mannosylation.

## DISCUSSION

Glycosylation is one of the most common posttranslational modifications of extracellular proteins. Although the precise role of protein glycosylation remains unknown, it is essential for the health and survival of cells. Accordingly, glycosylation enzymes have been suggested as targets for antimicrobials; however, similarities between fungal and mammalian glycosylation pathways have hindered these efforts against eukaryotic pathogens. Loss of O-linked protein mannosylation in *Histoplasma* resulted in viable *Histoplasma* cells *in vitro*; however, fitness in a mammalian host was severely attenuated. *Histoplasma* has only one representative each of the Pmt1, Pmt2, and Pmt4 families, of which only Pmt1 and Pmt2 are required for *Histoplasma* infection. Attempts to create Pmt1 and Pmt2, as well as Pmt2 and Pmt4, double mutants were unsuccessful (data not shown), suggesting that there are overlapping functions or protein substrates among the Pmt proteins. Synthetic lethality of double *PMT* mutants of *Cryptococcus* (28, 29) and *Saccharomyces* (30) also suggests overlapping functions. The protein substrate specificities of Pmt1, Pmt2, and Pmt4 enzymes have not been defined, but loss of either Pmt1 or Pmt2 function results in nearly identical phenotypes in *Histoplasma*, consistent with Pmt1 and Pmt2 acting as a complex, which has been suggested by genetic and biochemical studies of *Saccharomyces* (20). While the precise mannan configurations assembled on *Histoplasma* proteins are currently undefined, the significant reduction in mannose on extracellular proteins, as well as recapitulation of the phenotypes due to the depletion of Mnt1, the second enzyme in the mannosylation pathway, confirms the role of Pmt2 in the mannosylation of proteins. MS analysis of Cfp4 and the glycans liberated from this protein revealed glycans up to three units in length (Fig. 1D), although longer chains are likely to exist because of loss of hexose units through the beta elimination reaction (i.e., peeling). The glycosylated mucin-like region of Cfp4 contains at least 28 total hexoses (Fig. S2), but whether all sites are glycosylated and if they have identical glycan lengths cannot be determined, given 16 potential O-linked glycosylation sites within the peptide. Nonetheless, the biochemical and MS data indicate that *Histoplasma* O-linked glycosylation is largely homopolymeric, consisting of mannose similar to that observed in other fungi. Loss of O-linked mannosylation caused a reduction in the galactose content of the culture filtrate, which may suggest that some *Histoplasma* mannan structures could be capped by galactose (31).

O-linked mannosylation in other fungi has been associated with the formation and function of the fungal cell wall. Mutants lacking Pmt functions are variably sensitive to cell wall-disrupting agents (e.g., calcofluor white and Congo red), can display cell separation defects following mitosis (32, 33), and lack modification of cell wall glycan assembly proteins (e.g., β-glucan synthases and chitinases) (30, 34). *Histoplasma* yeast cells lacking Pmt2 function maintain general cell wall integrity, showing only a minor increase in sensitivity to Uvitex (a polysaccharide-binding dye). Nonetheless, the outer surface of the yeast cell wall was altered (Fig. 2), as electron microscopy suggested an increase in exposed mannoproteins. This counterintuitive result (more mannan exposure with the loss of O-linked mannosylation) suggests that there may be compensation by increased expression of proteins modified by N-linked glycans or by increased N-linked glycosylation of the affected proteins. The latter situation has been shown to occur in at least one cell wall protein in *S. cerevisiae*, in which the cell wall protein Ccw5 is N-linked glycosylated in the Pmt4 mutant but not in wild-type *S. cerevisiae* (35). The increased mannan exposure due to loss of O-linked mannosylation is also supported by the MR-dependent recognition of Pmt2-deficient yeast cells. Together with the decreased β-glucan exposure, these results indicate that proper organization of the fungal cell wall requires O-linked mannosylation of proteins, likely those proteins that contribute to hydrolysis or formation of cell wall polysaccharide linkages, as these enzymes are often glycosylated (36, 37).

Despite the altered cell wall and glycan recognition, phagocyte associations were not affected. This is not unexpected, as *Histoplasma* yeast cells primarily target β-integrins (i.e., CR3) (38–40) for stimulating phagocytic uptake and minimize recognition by signaling receptors (e.g., Dectin-1) (41). Investigation of phagocyte-centric aspects of *Histoplasma* pathogenesis revealed no defects to explain the substantial *in vivo* fitness attenuation of yeast cells deficient in O-linked mannosylation; there was no change in proinflammatory cytokines *in vitro* (data not shown) or *in vivo* (Fig. S6), and the survival of yeast cells in cultured polymorphonuclear leukocytes (PMNs) and macrophages was unaffected (Fig. S4). Furthermore, depletion of phagocyte populations *in vivo* did not rescue the attenuation, demonstrating that O-linked mannosylation is not required for defense against PMNs or macrophages.

O-linked protein mannosylation is necessary for thermotolerance of *Histoplasma* yeast cells. The elevated temperatures of mammals was postulated as a major restriction of the evolution of fungi as pathogens of mammals but widespread proliferation of fungi as pathogens of plants (42). Indeed, the ability to proliferate at 37°C is one of the classic virulence determinants of *Cryptococcus* (43). For *Histoplasma* infections, elevated temperature is both good and bad. Elevated temperature is an essential signal to trigger differentiation into the pathogenic program. This differentiation is necessary for the expression of virulence factors that enable yeast cells to survive host immune defenses (44). However, *Histoplasma* yeast cells must also be able to survive the elevated temperature of a mammalian host. While *Histoplasma* cells lacking O-linked mannosylation can maintain the pathogenic yeast state, they had significantly limited thermotolerance; wild-type *Histoplasma* yeast cells tolerated temperatures of up to 38°C *in vitro*, but strains deficient in O-linked mannosylation were arrested/dead at temperatures above 37°C (Fig. 5). Sensitivity to elevated temperatures also characterizes other non-thermally dimorphic fungal species with defects in O-linked mannosylation (28–30, 34, 45), although the threshold is not as narrowly defined as with *Histoplasma*. *Cryptococcus neoformans* Pmt2 and Pmt4 mutants both grew well at 30°C but were unable to grow at 37 and 39°C, respectively (28, 29). In addition, *C. albicans* Pmt1 and Pmt2 are required for growth at 42°C (34) and *Aspergillus fumigatus* conidiation is severely attenuated at 50°C when lacking Pmt1 (45). While some *Cryptococcus* and *Candida* mutants have severe fitness deficiencies in mouse models, the attenuation *in vivo* has not been conclusively linked to temperature sensitivity; many of these mutants have significant structural deficiencies, and loss of Pmt4 in *C. albicans* results in virulence attenuation but not in temperature sensitivity (34).

This study demonstrated that O-linked mannosylation of proteins confers sufficient thermotolerance to *Histoplasma* yeast to enable infection of mammalian hosts. Although *Histoplasma* infection stimulated the production of pyrogenic cytokines (i.e., IL-1β, IL-6, and TNF-α), pyrexia is not induced in mice. However, maintenance of a core body temperature of 37 ± 1°C was restrictive to *Histoplasma* yeast cells lacking O-linked mannosylation. Cyclophosphamide treatment artificially lowered the core body temperature, which rescued the *in vivo* fitness attenuation of yeast cells lacking Pmt2 function. Although cyclophosphamide affects multiple phagocyte populations, specific depletion of these cells did not rescue the attenuation, confirming that it is the cyclophosphamide-dependent reduction in body temperature that allowed Pmt2-deficient yeast cells to survive and proliferate in the host. Furthermore, reduction of the average core body temperature was required to permit the growth of O-mannosylation-deficient yeast cells, rather than prevention of fever, since treatment of mice with ibuprofen did not rescue the Pmt2 attenuation (data not shown). These findings, together with the perturbed organization of the cell wall, lead to the hypothesis that O-mannosylation maintains the stability and/or function of enzymes critical to the formation and integrity of the *Histoplasma* cell wall at elevated temperatures. Thus, O-linked mannosylation facilitates the thermotolerance of *Histoplasma* yeast cells and helps define how this thermally dimorphic fungus has become a successful fungal pathogen of mammals.

## MATERIALS AND METHODS

### *Histoplasma* strains and culture.

The *Histoplasma capsulatum* strains used in this study were derived from wild-type strain G217B (ATCC 26032) and are listed in Table S1. *Histoplasma* yeast cells were grown in *Histoplasma*-macrophage medium (HMM) (46) supplemented with 100 µg/ml uracil for growth of auxotrophs or with 25 µM FeSO_4_ for growth on solid medium.

10.1128/mBio.02121-17.7 TABLE S1  *Histoplasma* strains used in this study. Download TABLE S1, PDF file, 0.1 MB. Copyright © 2018 Garfoot et al. 2018 Garfoot et al. This content is distributed under the terms of the Creative Commons Attribution 4.0 International license.

10.1128/mBio.02121-17.8 TABLE S2  Cell wall sensitivity assay results. Download TABLE S2, PDF file, 0.01 MB. Copyright © 2018 Garfoot et al. 2018 Garfoot et al. This content is distributed under the terms of the Creative Commons Attribution 4.0 International license.

### Depletion of O-linked mannosylation.

*Histoplasma* yeast cells were mutagenized by *Agrobacterium*-mediated transformation (47), and the T-DNA insertion site was determined by thermal asymmetrical interlaced PCR (48, 49). The *pmt2*::T-DNA mutant (OSU129) was complemented with the *PMT2* locus (*PMT2* gene with 1,300 bp of the upstream sequence). Pmt1, Pmt2, Pmt4, and Mnt1 functions were depleted by RNAi (19). The RNAi vectors used were created in the *gfp*-sentinel vector (pED02) (7) by using 500 to 1,000 bp of the coding regions: *PMT1*, nucleotides (nt) 984 to 2045; *PMT2*, nt 1611 to 2113; *PMT4*, nt 931 to 1710; *MNT1*, nt 234 to 1055. Vectors were transformed into *gfp*-expressing sentinel strain OSU194 by *Agrobacterium*-mediated transformation, and sentinel green fluorescent protein (GFP) fluorescence was quantified with a modified gel documentation system (19) and ImageJ software (50).

### Immunoblotting analysis.

Culture filtrate proteins from wild-type (*PMT2*) or mutant (*pmt2*) yeast cells were treated with PNGase F (New England Biolabs) to remove N-linked glycans and then separated under reducing conditions by 10% SDS-polyacrylamide gel electrophoresis and transferred to nitrocellulose membranes. Proteins were detected with monoclonal antibodies to Cfp4 (clone 2D20) (21) and Sod3 (clone 3J23) and visualized with horseradish peroxidase (HRP)-conjugated anti-mouse antibody and HRP chemiluminescent substrate (Millipore).

### Saccharide composition analysis.

The saccharide content of extracellular culture filtrate proteins was analyzed by the alditol acetate method (51) after exchange into phosphate-buffered saline (PBS). *scyllo*-Inositol (an internal saccharide standard) was added, and the glycans from 50 µg of protein were hydrolyzed in 2 M trifluoroacetic acid (120°C for 3 h), followed by reduction with sodium borodeuteride (NaBD_4_) and acetylation by acetic anhydride. The analytes were separated by GC (Trace GC Ultra; Thermo Scientific) with a 30-meter nonpolar capillary column (Restek) (210 to 240°C at 2°C/min in 30 min) and analyzed by MS (DSQII; Thermo Scientific). Peaks corresponding to monosaccharides (glucose, mannose, and galactose) were identified, and the total amount of each sugar was calculated relative to the peak area of *scyllo*-inositol.

*O*-Glycan analysis of Cfp4 was performed by MS of glycans released from Cfp4 protein purified from *Histoplasma* yeast culture filtrates. A Cfp4:hexahistidine fusion protein lacking N-linked glycosylation sites (21) was overexpressed by *PMT2* or *pmt2 Histoplasma* yeast cells and purified from culture filtrates with HisPur cobalt spin columns (Thermo Scientific). Glycans associated with Cfp4 were released by nonreductive beta elimination and the addition of 1-phenyl-3-methyl-5-pyrazolone (PMP) (52). Briefly, 60 μg of ribose (as an internal standard) was added to 400 μg of Cfp4 and 0.5 M PMP and the samples were incubated at 50°C in 30% ammonia for 15 h. To recover the glycans, samples were extracted with CHCl_3_ and the aqueous layer was dried and resuspended in 5% acetonitrile and further purified with a C_18_ spin column (Pierce). Samples were analyzed with a MALDI Ultraflextreme mass spectrometer (Bruker) by using a matrix composed of 10:1 DHAP (2,6-dihydroxyacetophenone):DAHC (diammonium hydrogen citrate) mixed with oligosaccharide at 10:1. Samples were run in reflectron mode from 100 to 5,000 *m*/z.

### Cell wall sensitivity assays.

Yeast cells were grown in 96-well microtiter plates (53) with graded concentrations of the cell wall-destabilizing compounds Congo red (MP Biomedicals), SDS (Fisher), sodium chloride (RPI), and Uvitex 3BSA (54). Wells were inoculated with 2 × 10^6^ yeast cells/ml in HMM and incubated at 37°C. Turbidity was measured by determining the optical density at 595 nm (OD_595_), and IC_50_s were computed by nonlinear regression of the dose-response data.

### Infection of mice and determination of virulence *in vivo*.

C57BL/6 mice (Charles River, Inc.) were *Histoplasma* infected by intranasal delivery. Their lungs were collected at various time points and homogenized, and serial dilutions of the homogenates were plated on solid HMM to determine the fungal burdens (CFU counts). Measurement of murine body temperature was performed with a probe thermometer (RET-3 probe; Kent Scientific) at 1100 and 2300 h each day. *In vivo* cytokine production was quantified with a proinflammatory multiplex panel (Mouse Proinflammatory Panel 1; Meso Scale Diagnostics) on lung homogenates 24 and 48 h after *Histoplasma* infection.

Phagocytic cells were depleted *in vivo* by treatment with cyclophosphamide (150-mg/kg intraperitoneal [i.p.] injection; Sigma) versus PBS, liposomal clodronate (250 µg administered intranasally; Encapsula NanoSciences) versus a liposomal control, and anti-GR-1 (Ly6C/Ly6G) antibody (clone RB6-8C5 500 µg administered i.p. every 48 h; Bio-X Cell) versus an isotype control antibody (anti-keyhole limpet hemocyanin [KLH], clone LTF-2; Bio-X Cell). All treatments were administered 24 h prior to infection, and mice were given enrofloxacin (Bayer) *ad libitum* in drinking water at 250 µg/ml to prevent opportunistic bacterial infection. Phagocyte depletion was monitored by light microscopy with Wright stain (Sigma) on whole blood smears (for cyclophosphamide and antibody injections) or bronchoalveolar lavage fluid (for clodronate administration).

### Flow cytometry of infected lungs.

C57BL/6 mice (Jackson) were infected with 10^6^ Uvitex 2B-labeled (10 µg/ml; 5 min) yeast cells administered intratracheally. Single cell suspensions were made from harvested lungs with a 70-µm cell strainer and treatment with collagenase D (1 mg/ml; Roche) and DNase (50 U/ml; Roche). Leukocytes were enriched by density sedimentation (60%/40% Percoll, 20 min, relative centrifugal force of 600) and collection of cells at the interface. Approximately 10^6^ cells were stained for cellular markers and fixed with 4% paraformaldehyde (30 min). The markers included CD64-fluorescein isothiocyanate, CD45-peridinin chlorophyll protein-Cy5.5, SiglecF-phycoerythrin-Cy7, Ly6C-BV650, CD11c-BV786, CD90.2-allophycocyanin (APC), B220-APC, MHCII-A700, Ly6G-BUV395 (all from BioLegend), and Near IR live/dead stain (Thermo, Fisher). Cells were analyzed with an LSRII flow cytometer (BD Biosciences), and data were processed with FlowJo software (version 10.1).

### Macrophage association and receptor binding.

Binding of yeast cells to Dectin-1 was quantified with Dectin-1-expressing 3T3 fibroblasts (25, 55). Briefly, yeast cells were added to Dectin-1-expressing 3T3 cells (2 h at 37°C) at a multiplicity of infection (MOI) of 50:1 (yeast-to-fibroblast ratio). Nonadherent yeast cells were removed, and adherent yeast cells were released by hypotonic lysis of the Dectin-1-expressing 3T3 cells and plated on solid HMM for CFU counting. Competition for binding was assayed by preincubating Dectin-1-expressing 3T3 cells for 1 h at 37°C with laminarin (1 mg/ml).

MR-dependent association of yeast cells with macrophages was quantified by knockdown of MR from human MDMs (56). Peripheral blood mononuclear phagocytes (PBMCs) were isolated from the buffy-coat layer after Ficoll-Paque PLUS sedimentation of human peripheral blood (400 × *g* for 40 min at room temperature). MDMs were differentiated from monocytes (RPMI plus 20% autologous serum, 5 days). Day 5 PBMCs were transfected with MR-specific (GUGGUACGCAGAUUGCACGUU, AGUCCUUUCCGAUAUUUG, and AUUUAAAGUGGUGUUGCCC) or scrambled control small interfering RNAs (siRNAs) by using Amaxa Nucleofector (Amaxa Biosystems) and the Y010 nucleofection settings (56). After 48 h, MDMs were infected with *Histoplasma* yeast cells at an MOI of 1:1 (yeast-to-macrophage ratio) and unbound yeast cells were removed after 2 h. Associated yeast cells were quantified by hypotonic lysis of MDMs and plating on solid HMM for CFU counting. Depletion of MR by siRNA was confirmed by immunoblotting (SC-48758; Santa Cruz Biotechnology).

### Transmission electron microscopy (TEM).

*Histoplasma* yeast cells were fixed in 2% glutaraldehyde in cacodylate buffer (0.1 M cacodylic acid, pH 7.4), postfixed in 1% osmium tetroxide, rinsed with buffer, and embedded in 2% low-temperature-gelling agarose. One-cubic-millimeter blocks were incubated in 1% uranyl acetate for 90 min prior to being dehydrated in a series of graded ethanol washes. Samples were incubated in propylene oxide for 20 min and infiltrated with Eponate 12 resin. Seventy-nanometer sections were stained in 2% aqueous uranyl acetate and Reynolds lead citrate and observed with a transmission electron microscope (FEI Tecnai Spirit) at 80 kV.

### Ethics statement.

Animal experiments were performed in compliance with the National Research Council Guide for the Care and Use of Laboratory Animals and were approved by the Ohio State University (OSU) Institutional Animal Care and Use Committee (2007A0241). Human cells were obtained from healthy volunteers after Health Insurance Portability and Accountability Act research authorization and written informed consent were obtained in accordance with the Declaration of Helsinki. The human subject protocol was reviewed and approved by the OSU Biomedical Sciences Institutional Review Board (protocol number 2008H0242) under the OSU Office for Human Research Protections (Federalwide Assurance number 00006378).
