# Supplementary figures and images for: O-Mannosylation of Proteins Enables Histoplasma Yeast Survival at Mammalian Body Temperatures
Source: mBio. 2018 Jan 2;9(1):e02121-17. doi: 10.1128/mBio.02121-17 (PMC5750402; doi:10.1128/mBio.02121-17)

A

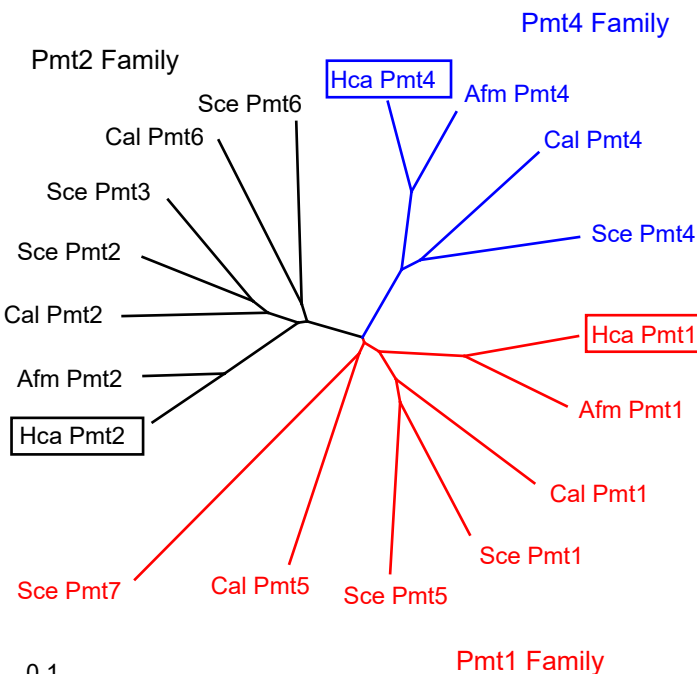

B

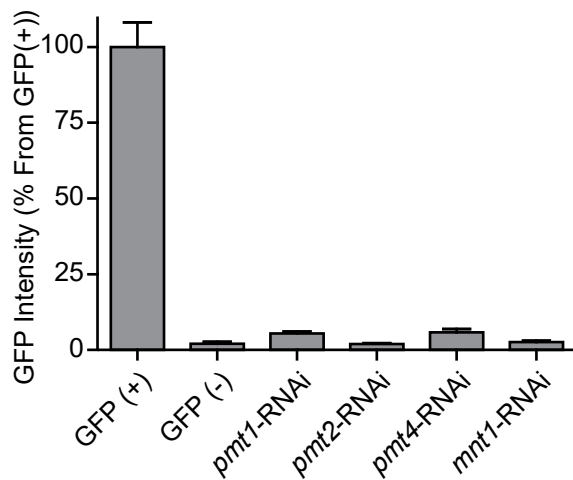

C

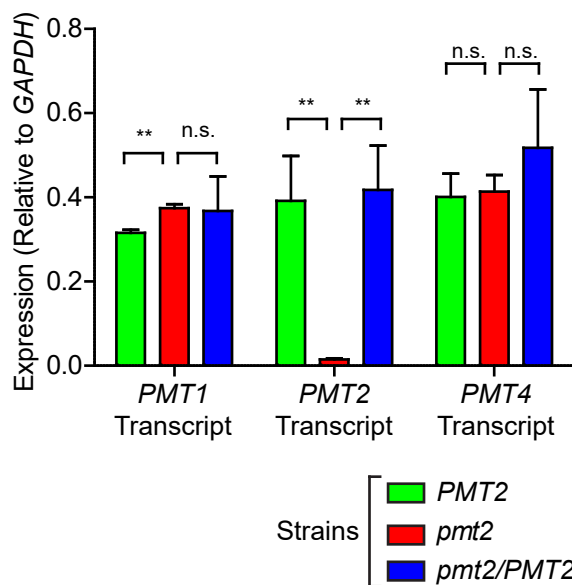

Supplement: FIG S1 [file mbo001183658sf1.pdf]

A

*PMT2*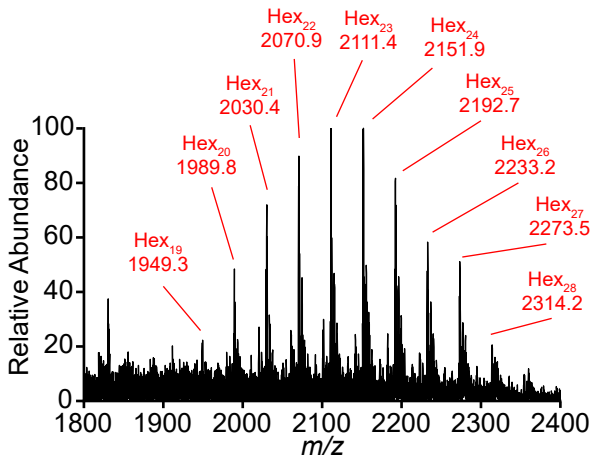AAVD**S**INGCLLG**S**NCPPPP**TTTTTT**PT**P**TP**P**TT**P**IT**P**ITPAAK

B

*pmt2*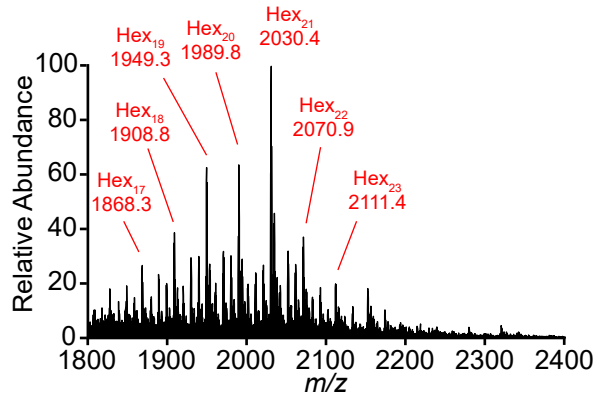

Supplement: FIG S2 [file mbo001183658sf2.pdf]

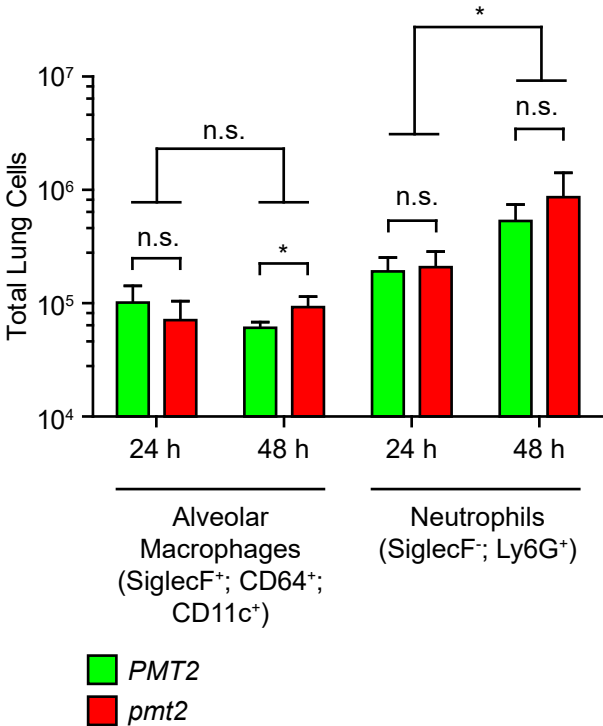

Supplement: FIG S3 [file mbo001183658sf3.pdf]

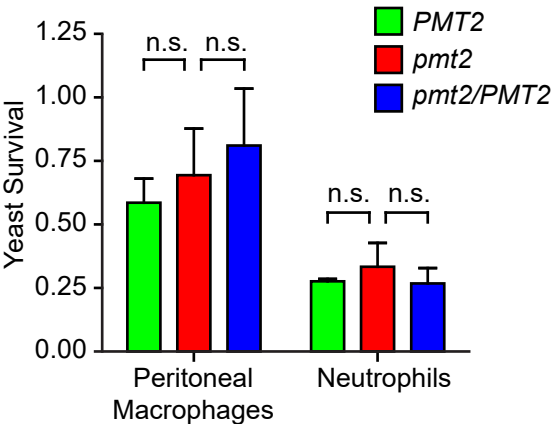

Supplement: FIG S4 [file mbo001183658sf4.pdf]

**A**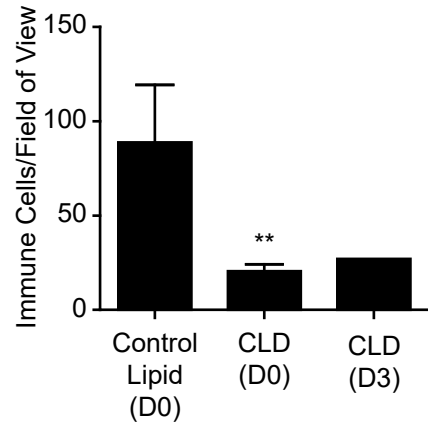**B**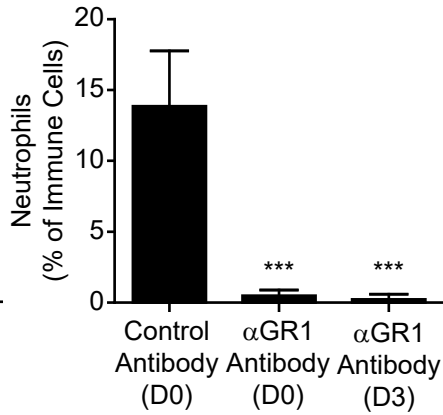**C**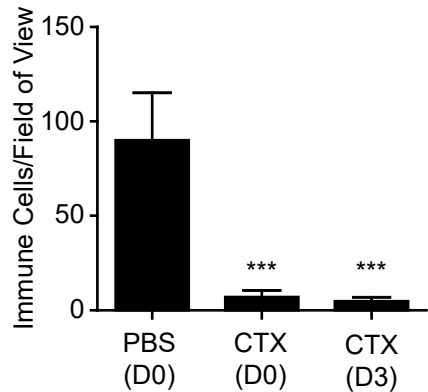

Supplement: FIG S5 [file mbo001183658sf5.pdf]

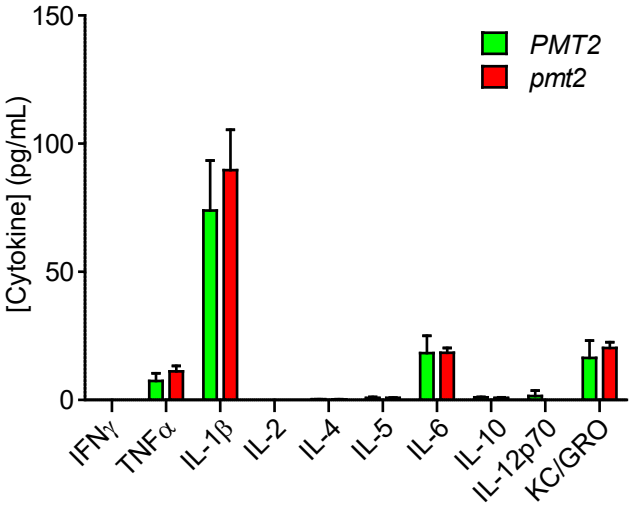

Supplement: FIG S6 [file mbo001183658sf6.pdf]
