# Supplementary material for: O-Mannosylation of Proteins Enables Histoplasma Yeast Survival at Mammalian Body Temperatures
Source: mBio. 2018 Jan 2;9(1):e02121-17. doi: 10.1128/mBio.02121-17 (PMC5750402; doi:10.1128/mBio.02121-17)
Supplement: TABLE S1 [file mbo001183658st1.pdf]

Table S1: *Histoplasma* Strains

Strain[1]	Genotype[2]	Other Designation
WU15	*ura5-42Δ*	
OSU129	*ura5-42Δ pmt2-1::T-DNA(hph)*	
OSU142	*ura5-42Δ zzz::T-DNA(pCR628: URA5, gfp)*	*PMT2*
OSU143	*ura5-42Δ pmt2-1::T-DNA(hph) zzz::T-DNA(pCR628: URA5, gfp)*	*pmt2*
OSU144	*ura5-42Δ pmt2-1::T-DNA(hph) zzz::T-DNA(pAG33: URA5, PMT2)*	*pmt2/PMT2*
OSU194	*ura5-42Δ zzz::pAG21* (*apt3*,*gfp*)	
OSU198	*ura5-42Δ zzz::pAG21* (*apt3*,*gfp*) *zzz::T-DNA(pED02: URA5, gfp-RNAi)*	*gfp*-RNAi
OSU199	*ura5-42Δ zzz::pAG21* (*apt3*,*gfp*) *zzz::T-DNA(pAG30: URA5, gfp:PMT1-RNAi)*	*PMT1*-RNAi
OSU201	*ura5-42Δ zzz::pAG21* (*apt3*,*gfp*) *zzz::T-DNA(pAG31: URA5, gfp:PMT4-RNAi)*	*PMT4*-RNAi
OSU203	*ura5-42Δ zzz::pAG21* (*apt3*,*gfp*) *zzz::T-DNA(pAG32: URA5, gfp:PMT2-RNAi)*	*PMT2*-RNAi
OSU287	*ura5-42Δ zzz::pAG21* (*apt3*,*gfp*) *zzz::T-DNA(pKG05: URA5, gfp:MNT1-RNAi)*	*MNT1*-RNAi
OSU330	*ura5-42Δ zzz:T-DNA(pCR624: URA5, rfp)*	PMT2
OSU331	*ura5-42Δ pmt2-1::T-DNA(hph) zzz:T-DNA(pCR624: URA5, rfp)*	pmt2
OSU364	*ura5-42*□ zzz::*pCR681 (URA5,* P_{H2B}*-CFP4:6xHIS)*	Cfp4(WT)
OSU365	*ura5-42*□ *pmt2-1::T-DNA* zzz::*pCR681 (URA5,* P_{H2B}*-CFP4:6xHIS)*	Cfp4(pmt2)

[1] strains were all constructed in the *Histoplasma* G217B background (ATCC 26032)
[2] gene designations:

zzz::T-DNA: T-DNA integration at an undetermined chromosomal location
apt3: aminoglycoside phosphotransferase (G418 resistance)
gfp: green-fluorescence protein
hph: hygromycin B phosphotransferase (hygromycin resistance)
rfp: red-fluorescence protein (tdTomato)
CFP4: culture filtrate protein
MNT1: mannosyltransferase
PMT: protein mannosyltransferase
URA5: orotate phosphoribosyltransferase
