# Supplementary material for: O-Mannosylation of Proteins Enables Histoplasma Yeast Survival at Mammalian Body Temperatures
Source: mBio. 2018 Jan 2;9(1):e02121-17. doi: 10.1128/mBio.02121-17 (PMC5750402; doi:10.1128/mBio.02121-17)
Supplement: TABLE S2 [file mbo001183658st2.pdf]

Table S2 – Cell wall sensitivity[1].

	Uvitex (m%)	Congo Red (uM)	SDS (m%)	NaCl (M)
PMT2	7.2±0.1	0.94±0.02	3.7±0.3	0.7±0.3
pmt2	5.7±0.7	0.67±0.02	3.2±0.7	0.4±0.2
pmt2/PMT2	8.9±0.8	0.90±0.01	3.5±0.3	0.5±0.4

[1] the mean concentration for 50% inhibition of yeast growth (IC_{50}) ± standard deviations among replicate assays (n=3) are listed
